# Supplementary material for: Effect of the COVID-19 pandemic on bike-sharing demand and hire time: Evidence from Santander Cycles in London
Source: PLoS One. 2021 Dec 2;16(12):e0260969. doi: 10.1371/journal.pone.0260969 (PMC8639062; doi:10.1371/journal.pone.0260969)
Supplement: S1 File — (DOCX) [file pone.0260969.s001.docx]

**Supplementary Material**

**Effect of the COVID-19 pandemic on bike-sharing demand and hire time:**

**Evidence from Santander Cycles in London**

S. Heydari, G. Konstantinoudis, A. Behsoodi

Section 1.

Nimble is available from R-nimble.org, the NIMBLE project web site. Specifically, we used the following libraries in R: Nimble (a package for performing MCMC in R) [1], coda (a package for summarising and plotting of MCMC outputs and convergence diagnosis) [2], and ggmcmc (a package for MCMC diagnostics) [3].

References:

[1] de Valpine P, Turek D, Paciorek C, Anderson-Bergman C, Temple Lang D, Bodik R. Programming with models: writing statistical algorithms for general model structures with NIMBLE. Journal of Computational and Graphical Statistics. 2017; 26:403-413. https://doi.org/10.1080/10618600.2016.1172487.

[2] Plummer M, Best N, Cowles K, Vines K. “CODA: Convergence Diagnosis and Output Analysis for MCMC.” R News. 2006; 6(1):7–11.

[3] Fernández-i-Marin X. “ggmcmc: Analysis of MCMC Samples and Bayesian Inference.” Journal of Statistical Software. 2016; 70(9):1–20. doi: 10.18637/jss.v070.i09.

Section 2.

Here, as an example, we display the results of the leave-one-year-out cross-validation for the year 2019 based on the data from 2010 to 2018.


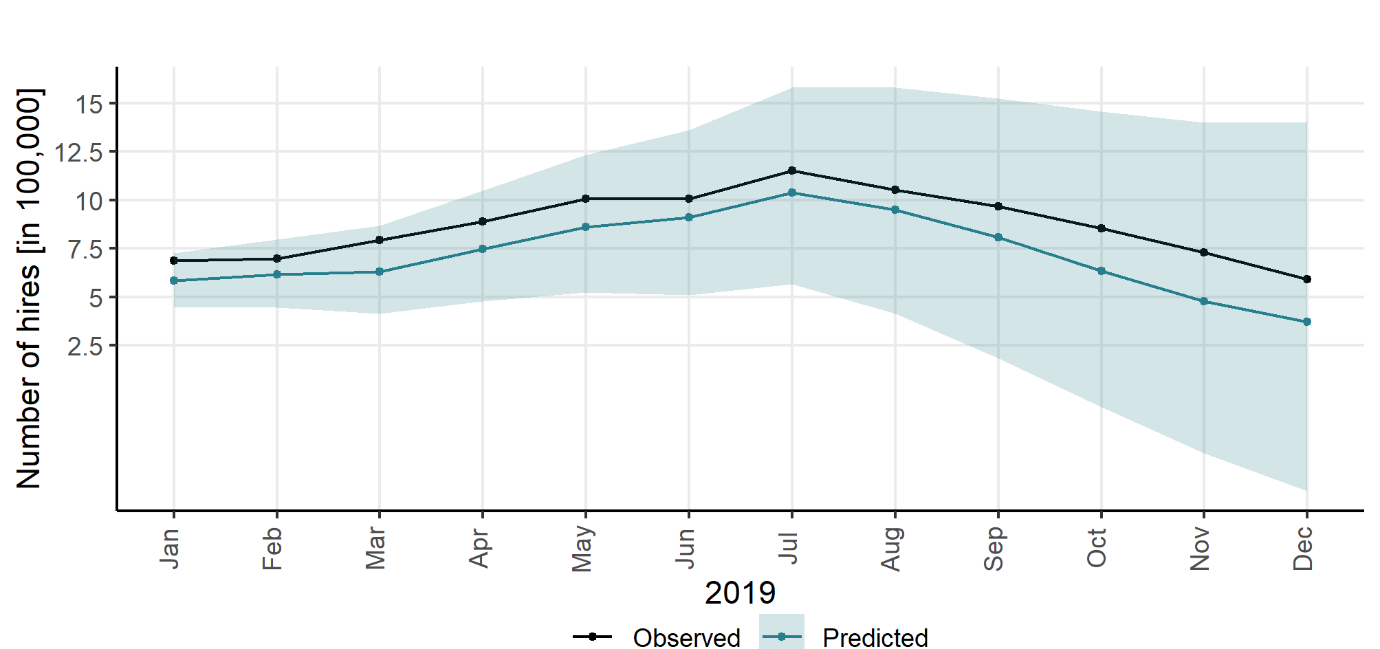


Fig 1 Observed vs. predicted hire numbers for the year 2019. Note: the shaded area indicates the 95% credible intervals around predicted values. See the electronic version for a colour view.


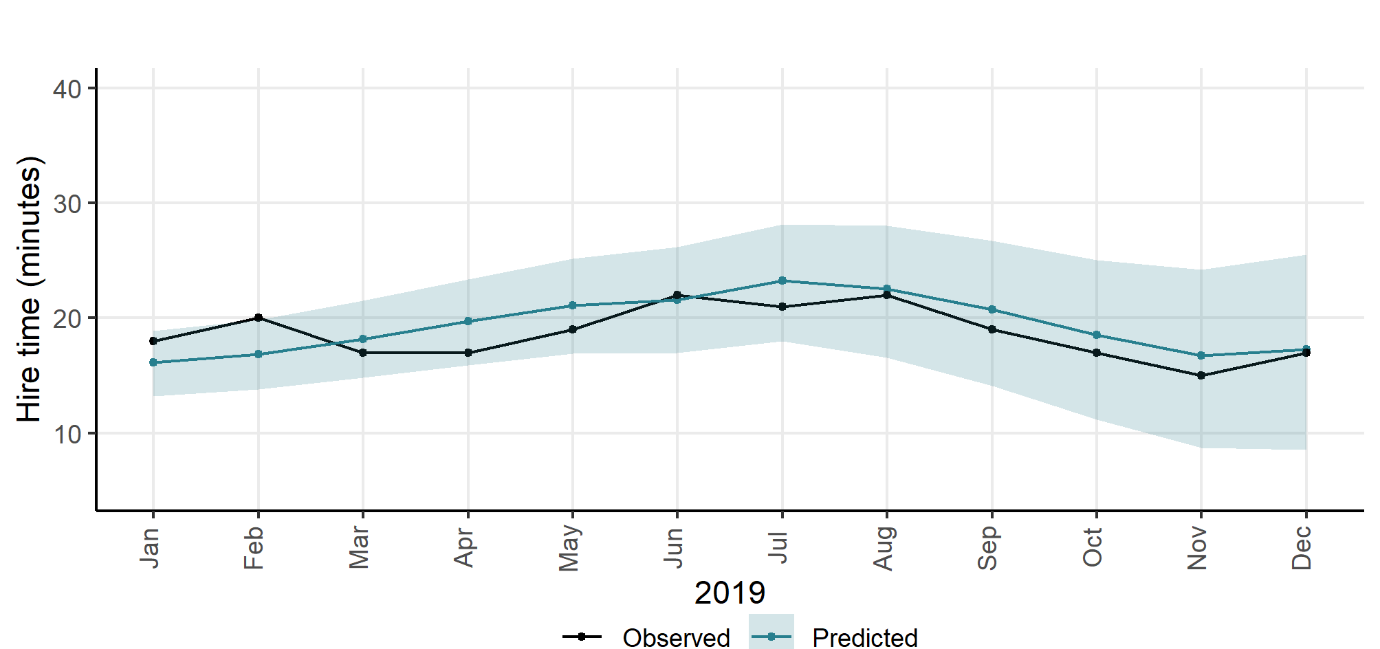


Fig 2 Observed vs. predicted hire time (trip duration) for the year 2019. Note: the shaded area indicates the 95% credible intervals around predicted values. See the electronic version for a colour view.
